# Identifying Acute Aortic Syndrome and Thoracic Aortic Aneurysm from Chest Radiography in the Emergency Department Using Convolutional Neural Network Models

**DOI:** 10.3390/diagnostics14151646

**Published:** 2024-07-30

**Authors:** Yang-Tse Lin, Bing-Cheng Wang, Jui-Yuan Chung

**Affiliations:** 1 Department of Emergency Medicine, Cathay General Hospital, Hsinchu Branch, Hsinchu 300003, Taiwan; qqyyog@gmail.com; 2 Department of Emergency Medicine, Sijhih Cathay General Hospital, New Taipei City 221037, Taiwan; 3 Department of Emergency Medicine, Cathay General Hospital, Taipei City 106438, Taiwan; 4 School of Medicine, National Tsing Hua University, Hsinchu 300044, Taiwan

**Keywords:** acute aortic syndrome, thoracic aortic aneurysm, convolutional neural network, emergency department

## Abstract

(1) Background: Identifying acute aortic syndrome (AAS) and thoracic aortic aneurysm (TAA) in busy emergency departments (EDs) is crucial due to their life-threatening nature, necessitating timely and accurate diagnosis. (2) Methods: This retrospective case-control study was conducted in the ED of three hospitals. Adult patients visiting the ED between 1 January 2010 and 1 January 2020 with a chief complaint of chest or back pain were enrolled in the study. The collected chest radiography (CXRs) data were divided into training (80%) and testing (20%) datasets. The training dataset was trained by four different convolutional neural network (CNN) models. (3) Results: A total of 1625 patients were enrolled in this study. The InceptionV3 model achieved the highest F1 score of 0.76. (4) Conclusions: Analysis of CXRs using a CNN-based model provides a novel tool for clinicians to interpret ED patients with chest pain and suspected AAS and TAA. The integration of such imaging tools into ED could be considered in the future to enhance the diagnostic workflow for clinically fatal diseases.

## 1. Introduction

Acute aortic syndrome (AAS) encompasses patients with a similar clinical profile, including penetrating atherosclerotic aortic ulcer, intramural aortic hematoma (IMH), and classic aortic dissection (AD) [1]. AAS presents a life-threatening medical condition, challenging prompt diagnosis in the emergency department (ED) due to its atypical presentation. The long-term survival rate among AD patients ranges from 30% to 60% [2]. Thoracic aortic aneurysm (TAA) also represents a medical emergency, with an overall mortality rate of 97% to 100% upon rupture [3]. Immediate surgical repair is necessary for all symptomatic TAAs, underscoring the importance of early diagnosis for successful treatment.

Despite advancements in medical technology over the centuries since AD was first reported, the mortality rate remains high, ranging from 18% to 25% [4]. This high mortality rate may be due to the fact that, clinically, AAS can lead to rapid deterioration and requires immediate detection and management [5]. Current diagnostic tools, including electrocardiography, chest radiography (CXRs), and symptom assessment, exhibit poor sensitivity and specificity for TAA diagnosis. CXRs, however, offers a convenient diagnostic tool in the early stages due to its immediate availability and low radiation dose. Radiographic signs of AD in CXRs include widening of the mediastinum, double or irregular aortic knob, left pleural effusion due to a hemothorax, and displacement of intimal calcification [6,7,8,9]. Nevertheless, some studies have noted that 37.4% of Type A AD cases show no mediastinal widening, and 12.4% exhibit no radiographic abnormalities [4].

Currently, computed tomography angiography (CTA) serves as the gold standard diagnostic tool for AD due to its high sensitivity (100%) and specificity (98–100%) [10,11]. However, CTA may be more time consuming than single CXR, has a risk of contrast medium injection, and is more expensive, and thus it is not an easily accessible screen image tool clinically.

Traditionally, clinical physicians diagnose AAS primarily based on symptoms and signs and then proceed with basic diagnostics such as CXR, electrocardiogram, blood tests, and focused ultrasound to differentiate it from other diseases. If AAS is highly suspected, CTA will be arranged for confirmation. However, sometimes the symptoms and signs are not specific, making early diagnosis challenging. With the introduction of artificial intelligence (AI) in CXR imaging, we can detect AAS at an earlier stage, potentially accelerating the diagnostic process and consequently shortening the time to treatment or surgery.

In recent years, AI interpretation of medical images has gained traction, offering objective interpretation, examination of a large number of image features, automation, rapid diagnosis, and continuous improvement through rigorous database establishment [12,13,14,15]. For example, deep learning networks have been utilized in medical image processing to aid in the prognosis and diagnosis of diseases such as breast cancer, lung cancer, and brain tumors [16]. A particular study highlighted that the ResNet-18 model demonstrates exceptional performance in characterizing dense tissue patterns, which is a critical risk factor for the development of cancerous cells in women’s breasts [17].

To date, AI applications in AD diagnosis have been limited to basic clinical data analysis (area under the curve, AUC: 0.857) [18] and computer tomography (CT) interpretation assistance [19]. Research on enhancing the efficiency of automated radiography interpretation remains scarce. Many medical imaging studies have employed existing public convolutional neural network (CNN) architectures for transfer learning, showing promising results in interpreting CXRs [20,21].

Reviewing the literature, most existing studies have focused on a single CNN model to predict AD via CXRs. However, research comparing different CNN models is lacking. Therefore, this study aims to utilize four different CNN-based models as objective imaging tools to expedite the diagnosis of aortic syndromes via CXRs. It also seeks to evaluate the performance of these models and discuss their potential application in the ED, with the expectation of improving the efficiency of CXR interpretation by emergency physicians and enhancing the accuracy of disease diagnosis.

Furthermore, this study also makes several contributions to the field of medical AI:

1. This study compares the performance of four various CNN architectures on medical imaging, demonstrating the varying effectiveness of different models in CXR diagnosis.
2. The ground truth for disease diagnosis in this study was established using CTA, which is the gold standard imaging tool for aortic disease.
3. By incorporating class activation mapping (CAM) in CXRs and comparing it with CTA, our study demonstrates that CNNs have the potential to accurately pinpoint aortic lesions.
4. This research highlights the specificity of CNNs in disease diagnosis, suggesting that they can be practically applied in emergency clinical settings.

## 2. Method

### 2.1. Study Design

This was a retrospective case-control study conducted in the ED of the Cathay Medical System (CMS), Taiwan, which comprises three hospitals: one medical center (located in Taipei), one regional hospital (located in New Taipei City), and one district hospital (located in Hisnchu City). The medical center has a capacity of 800 beds and an estimated annual ED visit volume of 60,000. The regional and district hospitals have capacities of 642 beds and 348 beds, respectively, with estimated annual ED visit volumes of 48,000 and 30,000. Patients who visited these three hospitals between 1 January 2010, and 1 January 2020, were enrolled in the study.

### 2.2. Data Collection and Assignment to Case and Control Groups

**Figure 1:**
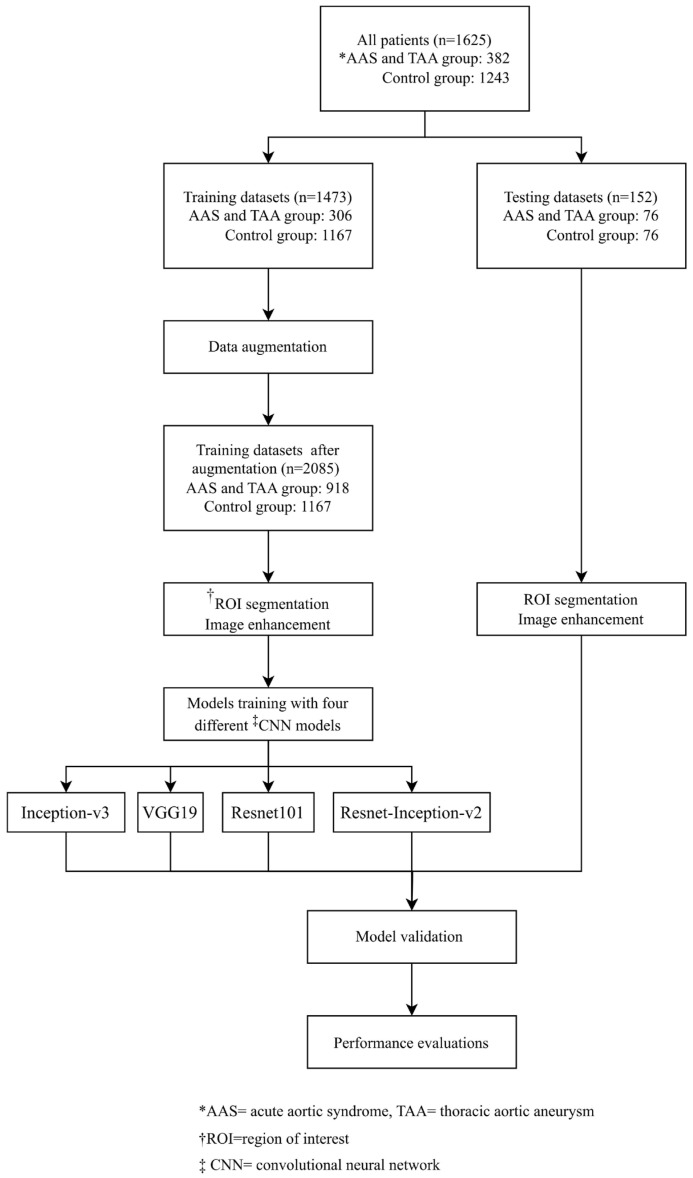
The flow chart of the study.

The experimental group included CXRs of patients who met the following criteria: (1) emergency patients who were older than 18 years old, with the chief complain of chest or back pain. (2) Patients underwent CXRs and computer tomography angiography in the ED. Patients in this group also fit the following image criteria: (1) diagnosed as AAS and IMH or (2) enlarged TAA (>5.5 cm) as confirmed by CTA. Exclusion criteria for the experimental group were: (1) trauma patients; (2) patients who had implants or a history of thoracic surgery; and (3) lesions on the CXRs, such as pneumonia, pulmonary edema, and tumors in the thorax cavity and mediastinum, as these lesions may interfere with the interpretation of the thoracic aorta. Other than chest images, patients’ gender, age, and underlying medical conditions were also collected. The underlying medical conditions of each patient were identified via the ICD-9 and recorded during the ED visit, including hypertension, atherosclerosis, renal insufficiency, ischemic heart disease, diabetes, and cerebral vascular disease, which can be related to aortic disease [22]. The control group included the CXRs of patients who met the following criteria: (1) emergency patients who were older than 18 years; (2) with a complaint of chest and back pain and who underwent CXR and computer tomography in the ED; (3) and whose CTA diagnosis report showed no evidence of AAS or TAA. The CTA images were interpreted and confirmed by board-certified radiologists. Gender, age, and underlying medical conditions were collected to analyze the characteristics of the control group patients. The patients’ demographic information was obtained from the electronic medical records of the CMS, while the images were sourced from the picture archiving and communication system (PACS) of the CMS. The flowchart of the study is illustrated in Figure 1.

### 2.3. Region of Interest

The ROI algorithm can extract specific signals from an image and maintain the anatomical structure we are interested in. It can maintain good image quality during the image decompression process of the CNN model. Therefore, such a method can prevent interference on the experimental results while accelerating the computational speed [23,24]. In order to train the ROI model, two hundred images were randomly selected from all image data, and the dataset was further divided into 80% for the training group (160 images) and 20% for the testing group (40 images). A board-certified emergency physician (the attending physician, who has 3 years of experience) manually annotated the training dataset to select the structures near the mediastinum, including the ascending aorta, aortic arch, descending aorta, and cardiac structure; After that, an aggregate channel features (ACF) object detector was used as the architecture for the ROI model training [25]. While detecting the CXRs automatically, the object detector model gave three sets of coordinates of ideal bounding boxes to show the rectangular area where the entire thoracic aorta may be located; the ROI was then defined as the intersection of the three ideal bounding boxes. The ROI selection step retained important structures in images while excluding other interfering factors such as text labels, abdominal images, neck bones, etc.

**Figure 2:**
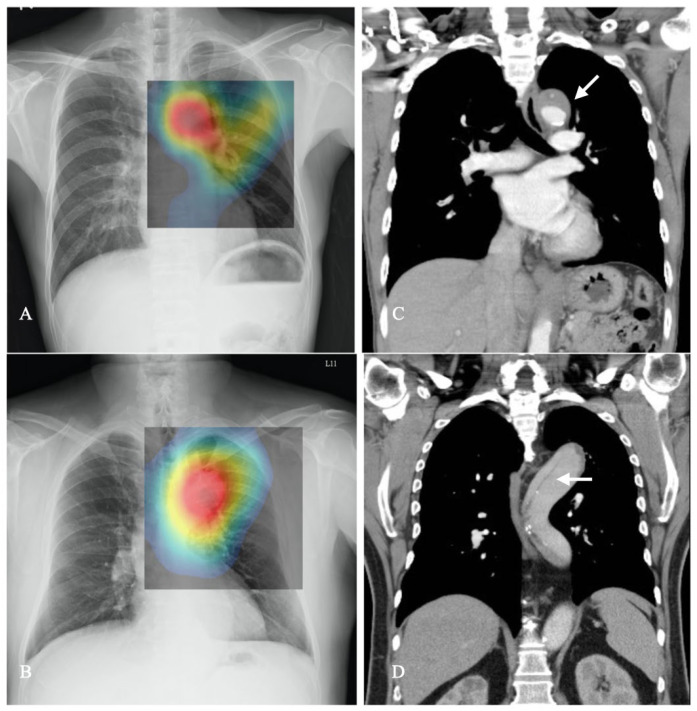
The images on the left (**A**,**B**) show the location of a possible aortic lesion on the CXR marked by the CNN model (VGG19), and these areas are colored by CAM to make it easy for users to interpret them. The pictures on the right (**C**,**D**) are the CTA images of the same patient showing the true location of the patient’s aortic lesion. After comparing with the CAM image, it was found that the CNN model correctly interpreted the location of the lesion on the CXRs, arrow: false lumen of aortic dissection.

Finally, in the testing dataset, emergency physicians manually interpreted the model, and the ACF object detector trained a model that could automatically detect the ROI. In the testing dataset, the ROI area consisting of the thoracic aorta structure was marked with an accuracy rate of 100% (40/40). This ACF model was then used in the next experiment to detect the ROI of different CXRs before proceeding to the next step of image processing. In Figure 2A,B, the central black square area on the chest image demonstrates the regions of the aorta and mediastinum automatically labeled by our ROI model.

### 2.4. Image Enhancement

**Figure 3:**
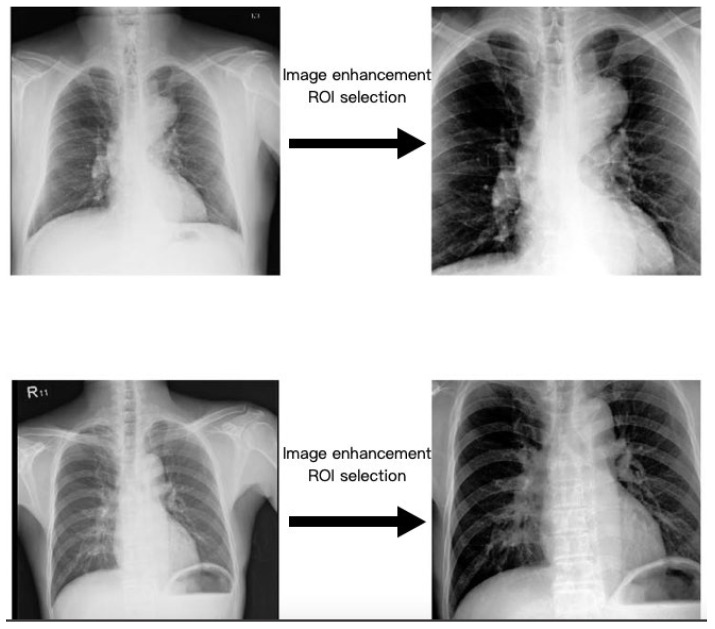
Image enhancement and ROI selection of original images for better image quality and normalization before model training.

This study utilized contrast limited adaptive histogram equalization (CLAHE), a technique widely employed in medical imaging [26]. CLAHE has been found to offer enhanced visualization of the mediastinal cavity and thoracic aorta without over highlighting high-density structures, such as bones, compared to other algorithms like histogram equalization and adaptive histogram equalization. Consequently, CXR images processed with CLAHE exhibit improved performance in training both the ROI model and the CNN model (Figure 3).

### 2.5. Data Augmentation

**Figure 4:**
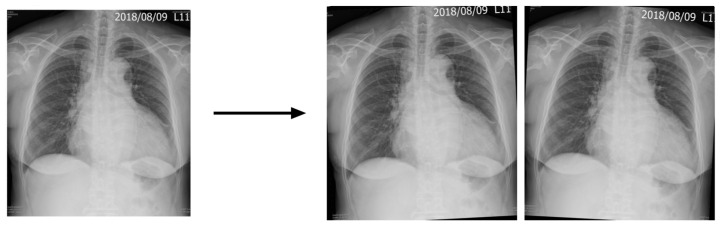
Image augmentation: original images were rotated by 3 degrees clockwise and counterclockwise.

Given the greater number of cases in the control group compared to the AAS and TAA groups (*n* = 1167 vs. 306), we augmented the images of patients with AAS and TAA in the training dataset to achieve data balance (*n* = 1167 vs. 918) (Figure 1). This augmentation involved rotating CXRs of the AAS and TAA groups in the training dataset. We decided to rotate these images (*n* = 306) by three degrees clockwise (*n* = 306) and counterclockwise (*n* = 306), and combined these images with the original CXRs (*n* = 306) to increase the data numbers (*n* = 918), as demonstrated in Figure 4.

### 2.6. Pre-Trained CNN Model

CNNs have found extensive application in deep learning, encompassing tasks such as image classification, object detection, and feature extraction. A CNN typically comprises various types of neural network layers, including convolutional layers, MaxPooling layers, dropout layers, fully connected layers, and SoftMax. Over time, CNN architectures have evolved, with many medical imaging studies leveraging existing public architectures for transfer learning.

In this research, we utilized four pre-trained CNN models (InceptionV3 [27], Inception-ResNet-v2 [28], VGG19 [29], and ResNet101 [30]), all derived from the ImageNet Database, which was originally developed for interpreting natural images. After image augmentation and preprocessing, we resized the images and inputted them into these four different CNN architectures for transfer learning to establish models. Our data were divided into training datasets (80% of all images) and testing datasets (20% of all images). We then assessed the performance of these models on the testing dataset by evaluating their ability to interpret CXRs.

### 2.7. Statistical Analysis and Model Performance Evaluations

After testing the four different CNN-based models in the testing dataset, the final result of their confusion matrix was acquired. The contents of the confusion matrix for each model were analyzed for their sensitivity (precision), specificity, precision, F1 score, and accuracy of the testing dataset. The F1 score is a popular tool for evaluating the performance of AI models. The F1 score represents the classifier’s performance in both precision and recall; thus, the F1 score was used in this study to decide which model was the most successful.

$$F1\;score=2\frac{precision\times recall}{precision+recall}$$

The area under the receiver operating characteristic (AUROC) was used to evaluate the performance of the classifier models. Without threshold selection and calculation of precision and recall, the AUC represents the overall diagnostic performance of an AI model and is widely used in the machine learning field [31]. CAM is used to evaluate and weight each CNN-based model while interpreting the CXRs [32]. Although the CNN model can handle non-linear and high-degree-of-freedom data (such as images), it lacks interpretability, as the model itself could not identify the relationship between input data and output results [33]. Therefore, researchers often need to further verify the performance of the CNN model in order to increase users’ confidence. CAM is currently an effective tool to visualize the featured maps extracted by CNN. In the research process, when it is necessary to adjust training parameters or select algorithm tools, CAM can help researchers to confirm whether the model’s prediction results are accurate and whether adjustments are clinically reasonable when evaluating the model. CAM can provide a reference for researchers to adjust parameters and avoid model over fitting. In clinical applications, the visualized image features of CAM can allow clinical users to quickly enter the situation, thereby increasing the willingness of clinicians to use the CNN model tool (Figure 2C,D).

### 2.8. Training Parameters, Software, and Hardware

The software used in this study was Matlab 2020b; it was used for image pre-processing, model training, and statistical analysis of the results. The parameters used in the training CNNs were as follows: optimization algorithm; Adaptive Moment Estimation (ADAM); mini batch size: 20; max epochs: 6; and initial learn rate: 0.0001. We used an Intel^®^ Core™ i7-10750H as the CPU (Central Processing Unit) and an AMD Radeon™ Pro 555X as the GPU (Graphics Processing Unit) for building and training our models.

## 3. Results

In total, 1625 patients’ CXRs were included in this study, with 382 patients in the AAS and TAA group and 1243 patients in the control group. Analysis of patient demographics revealed a higher percentage of males in the AAS and TAA groups compared to the control group, accounting for 64.4% and 58.5%, respectively. Furthermore, the AAS and TAA groups exhibited a higher mean age ± standard deviation of 69.5 ± 15.5 years, compared to 65.1 ± 17.0 years in the control group. Hypertension was more prevalent in the AAS and TAA groups, with 69.1% of patients affected, compared to 55.3% in the control group. Similarly, the prevalence of all underlying medical conditions was higher in the AAS and TAA group, with 77% of patients affected, compared to 70% in the control group (Table 1).

In terms of demographic distribution, patients in the AAS and TAA groups were older and had a higher proportion of hypertension, which was significant (*p* < 0.01), consistent with the known risk factors for AAS. Although the difference was not statistically significant (*p* = 0.04), the AAS and TAA groups had a higher proportion of male patients. Previous literature has shown that patients with emergency chest pain or those in the AAS and TAA groups exhibit a similar gender distribution.

Regarding dataset organization, approximately 80% of the images from both groups were randomly allocated to the training datasets, resulting in 306 images in the AAS and TAA group and 1167 images in the control group, while the remaining 20% of images were assigned to the testing datasets, with 76 images in each group. To ensure data balance in the training datasets, data augmentation techniques were employed, resulting in a total of 2085 images (918 in the AAS and TAA group, and 1167 in the control group) (Figure 1).

For dataset arrangement, approximately 80% of images from both groups were randomly selected for training datasets (AAS and TAA group: 306, control group: 1167) and the remaining 20% for testing datasets (AAS and TAA group: 76, control group: 76). Data augmentation was used to increase the number of images to 2085 (AAS and TAA group: 918, control group: 1167) in order to achieve data balance in our training datasets.

**Figure 5:**
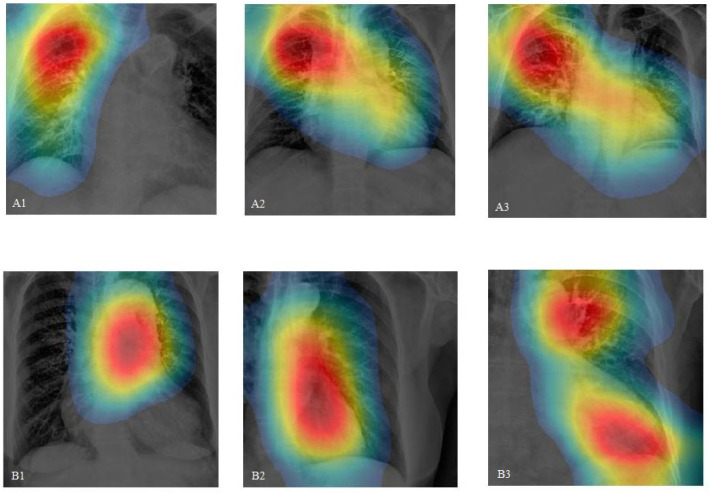
(**A1**–**A3**) The CAM images of the original CXRs interpreted by the CNN model show that some feature extraction areas are not logically related to the aortic structure. In contrast, (**B1**–**B3**) displays the results after the ACF-generated feature extraction model processed the images. Training the CNN model with ROI-focused images shows that the model focuses more on the anatomical structures of the mediastinum, without being distracted by surrounding organs.

During algorithm development, images processed without ROI selection via the ACF model sometimes caused the CNN model to lose focus, as shown in Figure 5(A1–A3). Conversely, images processed with ROI selection allowed the CNN to concentrate on the true anatomical structures, such as the aortic arch and thoracic aorta (Figure 5(B1–B3)), enhancing the clinical reliability of the CNN model.

We trained the processed images using four different CNN architectures for CXR interpretation and validated each model using the testing datasets (AAS and TAA group: 76, control group: 76). Analysis of model performance revealed that the Inception-v3 model attained the highest F1 score of 0.76, indicating strong overall performance. The VGG19-based model achieved the highest AUC of 0.84, highlighting its robust discrimination ability between classes. Both models demonstrated similar specificity, with the VGG19 model and the Inception-v3 model achieving specificities of 88%. Additionally, both the Inception-v3 and VGG19 models exhibited superior precision, at 85% and 82%, respectively, demonstrating their ability to correctly identify positive cases.

In contrast, the Resnet101 model showed lower sensitivity at 64%, suggesting it may miss more true positive cases compared to Inception-v3 and VGG19. It also had a lower AUC of 0.68, indicating less effective discrimination between classes compared to the other models. The Res-net-Inception-v2 model demonstrated consistent but modest performance across all models, with sensitivity, specificity, precision, and F1 scores all around 64%.

These findings highlight the varying effectiveness of different CNN architectures in the task of X-ray interpretation, with Inception-v3 and VGG19 outperforming Resnet101 and Res-net-Inception-v2 in key metrics (Table 2).

To enhance the model’s reliability, we also collected digital CTA images of AAS and TAA patients and retrospectively compared them with CXRs. This was to determine whether the CNN model could not only correctly identify abnormal images but also pinpoint the lesion’s location. CAM was applied to the original CXRs to visualize the feature maps extracted by the CNN models, enabling the localization of possible aortic lesions on CXRs and comparison with the same patients’ aortic CTA images (Figure 4). This confirmed that the CNN has the potential to locate and visualize lesions, providing valuable diagnostic information to clinicians.

## 4. Discussion

The novelty of this study lies in the comparative analysis of four distinct CNN-based models to determine their diagnostic performance for AAS and TAA based on CXRs in the ED. Specifically, the study provides a comprehensive evaluation of four pre-trained CNN models (Inception-v3, VGG19, Resnet101, and Resnet-Inception-v2) across several key metrics, including sensitivity, specificity, precision, F1 score, accuracy, and AUC. The results showed that Inception-v3 had the highest F1 score, while VGG19 had the highest AUC. These findings align with a recent study using the ResNet18 CNN model to detect acute thoracic AD based on CXRs, which achieved a diagnostic accuracy of 90.20%, a precision of 75.00%, a recall of 94.44%, and an F1 score of 83.61% [34]. Another study explored the use of explainable AI methods like Grad-CAM to interpret the decisions of a CNN model for detecting aortic elongation, which can be an indicator of AD, from CXR images [35]. However, the algorithms, data sources, and study designs of these studies varied. Therefore, we have included a table that summarizes the comparison of three studies (current study and two recent studies [34,35]) on the use of CNNs for aortic disease detection in CXR images, detailing their case numbers, data sources, CNN types used, and targeted abnormalities in Table 3.

In current practice, the diagnosis of AAS and TAA is challenging and often overlooked by physicians in the ED due to several factors. Firstly, crowded EDs result in physicians having insufficient time to diagnose a patient [36]. Secondly, using CXRs to diagnose AAS and TAA lacks clear image feature definition, leading to subjective interpretation and inter-rater differences. Thirdly, AAS may present with atypical symptoms that can be easily overlooked. Fourthly, novice doctors, such as post-graduate-year doctors or junior residents, may be unfamiliar with the disease’s characteristics. Finally, fatigue from long working hours or night shifts can contribute to diagnostic errors.

The CNN models used in the present study offer significant advantages by automatically screening all CXRs to identify patients who may potentially have AAS or TAA, especially in crowded EDs where physicians may have limited time to diagnose a patient. CNN models also provide more objective judgment, are not constrained by past research, and may identify new features of AAS on CXRs. CNN models are also helpful for novice doctors who are not familiar with the disease’s characteristics. Unlike human physicians, CNN models are not affected by long working hours or fatigue. Moreover, the prompt diagnosis of AAS and TAA by CNN models allows ED physicians to initiate treatment before receiving the official radiology report from radiologists [37], thereby potentially enhancing the quality of medical care and prognosis. In Taiwan, several studies have reported using CNN models for interpreting X-rays [38], particularly for severe diseases that are easily missed in the ED, such as acute epiglottitis [39]. Furthermore, the combination of CAM technology demonstrated in this study (Figure 2) illustrates the CNN model’s ability to accurately locate aortic lesions during diagnosis, further highlighting its utility as a valuable medical assistance tool.

While developing diagnostic AI models for medical images, the preprocessing of images is as crucial as selecting the appropriate CNN structure. Preprocessing images using suitable image enhancement algorithms and selecting an ROI segmentation model are vital in our study settings. Image preprocessing can expedite the CNN model training process and enhance diagnostic accuracy [23,24,26]. However, employing CNN models directly on medical images may not suffice, as real-world medical images often contain considerable noise. Noise reduction can be achieved by combining various algorithms at different stages of image preprocessing, enabling the CNN model to focus on relevant information in CXRs. In this study, the ACF detector was initially utilized as the ROI segmentation model to identify structures such as aortic vessels, heart borders, and the mediastinum. CLAHE was then applied for image enhancement, enhancing the density of these soft tissue structures. Subsequently, these images were fed into various CNN architectures for training. Throughout the training process, CNN model parameters were adjusted based on prediction results and the CAM analysis. The training outcomes were further deliberated by clinical physicians and programming engineers, and our group determined which algorithms to select and how to adjust the parameters accordingly.

Patients presenting with chest pain represent approximately 10% of total ED visits. While most of these patients may be discharged following evaluation, a subset may ultimately receive diagnoses of acute and potentially fatal conditions such as acute coronary syndrome and AAS [40]. Our study findings demonstrate that both Inception-v3 and VGG19 exhibited superior performance compared to other methods, with Inception-v3 achieving the highest scores for sensitivity, F1 score, and accuracy. VGG19, while slightly behind Inception-v3 in these areas, showed the highest AUC, indicating its strong discriminative ability. Resnet101 and Resnet-Inception-v2, although performing adequately, lagged in several key aspects, suggesting room for improvement in these models for detecting AAS and TAA from CXRs in EDs (Table 2). The high precision and accuracy of Inception-v3 offers a critical role in an ED setting where timely and accurate diagnosis can significantly impact patient outcomes. The high precision (85%, 95% CI: 68, 100) indicates that when the model predicts a positive case, it is likely to be correct, which is important for maintaining the credibility of the diagnostic tool among clinicians. The accuracy (78%, 95% CI: 65, 92) shows its overall effectiveness in correctly classifying both positive and negative cases. Therefore, Inception-v3 would be the recommended model for use in the ED for screening AAS and TAA from CXRs.

There are several limitations to this study. Firstly, despite fine model adjustment and image preprocessing, the sensitivity of the CNN model to identify AAS and TAA remains at 68%. Previous studies have also noted the limitations of CXRs in diagnosing aortic diseases [4]. This may be attributed to the inherent constraints of CXRs, which typically provides flat, binary-colored, and single-view images, making it challenging to diagnose vascular diseases such as AAS and TAA, which have complex 3D anatomical structures. In fact, delving into the detailed structure or blood flow changes in aortic dissection requires the aid of advanced imaging tools. For example, one study utilized computational fluid dynamics (CFD) to model blood flow in aortic dissection, enhancing the understanding of hemodynamics in these cases [41]. Another review article introduced the application of image-based CFD simulations and four-dimensional flow magnetic resonance imaging (4D-MRI) to predict risks in patients with aortic dissection [42]. These cutting-edge imaging tools provide valuable insights for identifying, managing, and treating aortic conditions.

In further exploration of this topic, we suggest considering the combination of CNN models with these advanced imaging tools (CFD, 4D-MRI, and 3D reconstruction techniques) and expanding the image database for CNN model training. Additionally, integrating CNN models for image analysis with other machine learning models that utilize clinical information and parameters such as age, risk factors, symptoms, and vital signs could form a multimodal solution to complement the limitations of a single CNN model.

Secondly, this study is retrospective in nature, which inevitably introduces some selection bias in patient selection and grouping, despite efforts to mitigate this bias in the study design. However, such selection bias is a common challenge encountered in similar AI medical research during the developmental stages. Therefore, based on our findings, we anticipate the emergence of more prospective studies in the future, focusing on the application of AI in real-world medical scenarios.

## 5. Conclusions

A CNN-based model (Inception-v3) can provide good diagnostic performance for AAS and TAA based on CXRs, as indicated by its F1 score of 0.76, demonstrating a good balance between sensitivity and precision. Furthermore, the use of this CNN-based model may help physicians speed up the diagnosis process and avoid possible misdiagnoses, resulting in a non-invasive, objective, accurate, and fast diagnostic tool for AAS and TAA patients before they undergo CTA examination.

## Figures and Tables

**Table 1:** Patient characteristics.

Patient Characteristic	AAS and TAA Groups (*N* = 382)	Control Group (*N* = 1243)	*p* Value
Sex, *n* (%)			0.04
Male	246 (64.4)	727 (58.5)	
Female	136 (35.6)	516 (41.5)	
Age-yr (Mean ± SD)	69.5 ± 15.5	65.1 ± 17.0	<0.01
Underlying medical condition, *n* (%)	294 (77.0)	874 (70.3)	0.01
Hypertension	264 (69.1)	689 (55.4)	<0.01
Atherosclerosis	57 (14.9)	251 (20.2)	0.02
Renal insufficiency	63 (16.5)	205 (16.5)	0.10
Ischemic heart disease	77 (20.2)	378 (30.4)	<0.01
Diabetes mellitus	69 (18.1)	391 (31.5)	<0.01
Cerebral vascular disease	81 (21.2)	203 (16.3)	0.03

AAS, acute aortic syndrome; TAA, thoracic aortic aneurysm; *n*, number; SD, standard deviation.

**Table 2:** Performance of AD detection in AAS and TAA predictions using different CNN models.

CNN Pre-Trained Models	Sensitivity (%, 95% CI)	Specificity (%, 95% CI)	Precision (%, 95% CI)	F1 Score (95% CI)	Accuracy (%, 95% CI)	* AUC
Inception-v3	68 [50, 86]	88 [75, 100]	85 [68, 100]	0.76 [0.58, 0.91]	78 [65, 92]	0.82
VGG19	56 [39, 73]	88 [73, 97]	82 [66, 92]	0.67 [0.53, 0.80]	72 [61, 83]	0.84
Resnet101	64 [45, 83]	68 [50, 87]	67 [48, 86]	0.65 [0.46, 0.84]	66 [58, 89]	0.68
Resnet-Inception-v2	64 [45, 83]	64 [45, 83]	64 [45, 83]	0.64 [0.45, 0,83]	64 [45, 83]	0.67

* Area under the curve (AUC) and area under the receiver operating curve (AUROC).

**Table 3:** Comparison of this study with two recent studies on the use of CNN in aortic disease CXR research, including methods and results.

	This Study	Ribeiro et al. (2024) [35]	Lee et al. (2023) [34]
Case numbers	*N* = 1473 (normal= 1167, AAS and TAA= 306)	*N* = 8752 (Aortic elongation = 2350, non-aortic elongation = 6402)	*N* = 3331 (Positive images = 716, negative images = 2615)
Data source	Three hospitals: one medical center, one regional hospital, and one district hospital	VinDr-CXR dataset	Three tertiary academic hospitals
CNN type	Inception-v3 VGG19 Resnet101 Resnet-Inception-v2	DenseNet 121 EfficientNet B4	ResNet 18
Abnormalities targeted	AAS and TAA	Aortic elongation	Acute thoracic aortic dissection
Disease label	CTA report	Interpretation by radiologist	CTA report and surgery record
Image augmentation protocol	Rotation	Rotation, horizontal flipping, and vertical flipping	Rotation, horizontal flipping, and vertical flipping
ROI model architecture	Aggregate channel features object detector	* UNet	UNet
Image enhancement	CLAHE	Histogram equalization	Histogram equalization
CAM application	Yes, and comparison with patients’ CTA image	Yes	Yes

* UNet is a convolutional network architecture for fast and precise segmentation of images.

## Data Availability

All data generated or analyzed during this study are included in this manuscript.
